# Bridging Generations Through Movement: “How and Why” Intergenerational Programs Operate—A Systematic and Narrative Review

**DOI:** 10.3390/geriatrics9060139

**Published:** 2024-10-22

**Authors:** Giulia Di Martino, Carlo della Valle, Marco Centorbi, Andrea Buonsenso, Giovanni Fiorilli, Claudia Crova, Alessandra di Cagno, Giuseppe Calcagno, Enzo Iuliano

**Affiliations:** 1 Department of Medicine and Health Sciences, University of Molise, 86100 Campobasso, Italy; giulia.dimartino21@gmail.com (G.D.M.); carlodv95@gmail.com (C.d.V.); marco.centorbi@hotmail.it (M.C.); andreabuonsenso@gmail.com (A.B.); fiorilli@unimol.it (G.F.); giuseppe.calcagno@unimol.it (G.C.); 2 Department of Neurosciences, Biomedicine and Movement, University of Verona, 37314 Verona, Italy; 3 Department of Movement, Human and Health Sciences, University of Rome “Foro Italico”, 00135 Rome, Italy; claudia.crova@uniroma4.it; 4 Department of Human Sciences, Guglielmo Marconi University, Via Plinio 44, 00193 Rome, Italy; 5 Faculty of Medicine, University of Ostrava, 73000 Ostrava, Czech Republic; enzo.iuliano@uniecampus.it; 6 Faculty of Psychology, eCampus University, 22060 Novedrate, Italy

**Keywords:** healthy aging, elderly, children, adolescent, social interaction, exercise, healthy lifestyle, well-being

## Abstract

Well-being and social interaction are among the primary goals to be achieved for the elderly. Intergenerational physical activity (PA) has gained increasing attention due to its potential to encourage PA and social interaction, providing both social and physical benefits to both younger and older individuals. This review aimed to gain a deeper understanding of the potential roles of PA in facilitating intergenerational interactions and provide practical insights. **Methods:** Following PRISMA guidelines, the systematic review identified specific keywords to search for articles that met the chosen inclusion and exclusion criteria (n. 5 RCT articles, selected between 2009 and 2024), conducted by three independent reviewers. Scopus, PubMed, EBSCOhost, and Web of Science were consulted to identify relevant articles. Risk of bias was assessed using Cochrane RoB 2. For the narrative dissertation, articles were identified across three key areas of focus: types of PA, age groups, and intended goals. **Results:** Few studies have specifically implemented PA protocols in intergenerational relationships, and most have planned remote activities without monitoring outcomes. The main advantages of intergenerational PA are oriented towards the social and relational sphere rather than simple PA involvement. **Conclusions:** For the elderly, these programs may help mitigate age-related deficits, while children and adolescents, when adapting to their older counterparts, experience greater effectiveness when provided with clear guidance during shared activities. Considering the characteristics and needs of individuals of different ages, different activities must be proposed to obtain different results. The organization of workshops and preparatory sessions will help in facilitating relationships and interactions among participants.

## 1. Introduction

Well-being and social interaction are two of the main goals to achieve for the elderly. Increased engagement in social activities on a regular basis and a greater perception of social support were linked to enhanced cognitive functioning [1]. A higher level of social involvement in old age has been correlated with improved psychological well-being. However, this association does not indicate a straightforward cause-and-effect relationship, and the strength of the correlation may vary across different aspects of social engagement [2,3]. Older age implies a slowdown in social interactions that leads to isolation and loneliness, while intergenerational interactions can result in an opportunity to keep up with new habits and customs typical of the life of new generations [4]. In recent years, intergenerational PA programs, based on the intergenerational theory, which draws upon elements from relationships, activity, cultural transmission, reciprocal transformation, and the alignment of needs and developmental assets [5,6], have gained increasing attention due to their potential to encourage PA and social interaction, providing both social and physical benefits to both younger and older individuals [7,8]. 

Actively engaging in group PA has proven successful in averting falls, addressing chronic conditions and disabilities, and promoting overall well-being among elderly individuals. A beneficial link between generations during PA experiences could offer added advantages in terms of physical and mental well-being for both age groups. There are mutual benefits when children were partnered with older adults [9]. The Intergenerational programs show the potential to preserve social capital by recognizing older adults as cultural, historical, and educational values. Elderly people’s expertise-derived abilities contribute to their roles as mentors, guides, and exemplars for younger generations [10]. The presence of younger individuals helps older adults stay socially connected and feel useful by allowing for the use of their experience to positively impact youth education and development. Intergenerational interactions enhance physical, cognitive, and social activity for both age groups. Studies show that while youth benefit educationally, older adults also gain from exchanging attitudes across ages [11]. This partnership could be a key to creating self-sustaining, cost-effective, and successful programs [12]. Moreover, if the intergenerational program includes PA, both groups may increase their amount of daily active time, following the recommended guidelines for each age group, considering that most older adults become physically inactive over the years incurring into health-related issues [13,14].

To attain these benefits, engagement in intergenerational social and physical activities should be frequent and long-term, and properly organized to be enjoyable for both older adults and younger participants, promoting adherence. Fun, motivation, attractiveness, perception of benefits should be the key to the structure of intergenerational physical activity programs [15].

How and why these programs are organized results in positive engagement, especially for older adults, according to their abilities and desires [16]. 

Therefore, the purpose of this systematic and narrative review was to investigate the connection between the type of physical activity, age group, and the intended goals of intergenerational programs, while also providing practical insights.

## 2. Materials and Methods

The Preferred Reporting Items for Narrative and Systematic Reviews (PRISMA) 2020 guidelines [17] were followed in the preparation, conduct, and presentation of the systematic review. For this systematic review, a protocol was registered on the PROSPERO platform. The present manuscript also includes a narrative review that analyses different aspects related to cognitive benefits, as well as motor, physical well-being, and social–emotional benefits associated with intergenerational training. 

### 2.1. Information Sources and Search Strategy

To identify relevant articles, the following online databases were used: Scopus; PubMed, EBSCOhost, Web of Science. In addition, citations, and reference lists of retrieved relevant reviews, were screened for any further studies.

The search was conducted in July 2024. The database was queried to search the following terms in the articles’ title, abstracts, and keywords: (“Intergenerational” OR “Cross-generational” OR “Multi-age” OR “Multigenerational” OR “Parenting”) AND (“Physical activity” OR “Exercise” OR “Training”).

### 2.2. Eligibility Criteria for the Systematic Review

The studies were looked into using the PICOS method as reported in Table 1. Papers were included if they met the following criteria: (a) Randomized Controlled Trial; (b) a total sample size of at least 30 participants; (c) protocols based on intergenerational PA; (d) intergenerational PA between children and adults, adolescents and adults, children and older adults, or adolescence and older adults; and (e) articles published from 2009 July to 2024 July.

Exclusion criteria: (a) not written in the English language; (b) intergenerational interventions with lack of assessment or monitoring on the performed PAs; (c) study projects that did not report outcomes; (d) protocols that did not effectively reflect intergenerational activities, such as PAs carried out during pregnancy; (e) studies that are commentaries, editorials, or congress excerpts. 

The databases were queried by two independent researchers. After removing duplicates, the two researchers independently evaluated the compliance of each report with the inclusion and exclusion criteria. When the two researchers disagreed, a third researcher was consulted to decide whether to include or exclude the report. The reports were first screened based on the title, then on the abstract and finally on the full paper. After the study selection, the following data were extracted from each eligible study: characteristics of studies (authors, year, study design, and country), sample size characteristics (sex, age), type of intervention and duration of the study, and outcome data (cognitive benefits, motor, physical well-being, and social–emotional benefits, overcoming social barriers). When studies reported their data only in graphical form, this was converted into metric data.

### 2.3. Eligibility Criteria for the Narrative Review

The narrative review focused on studies examining intergenerational physical training or activities, emphasizing interactions across various age groups, including children, adolescents, adults, and the elderly. The review included study designs, such as cohort studies, case studies, and qualitative research, to have a comprehensive overview. Different generations, both younger (children, adolescents) and older (adults, elderly) were included. Outcomes reported in the articles were based on cognitive benefits, motor, physical well-being, and socio-emotional benefits resulting from intergenerational physical training. Considered articles were published from 2009 July through to 2024 July.

The review examined different physical training interventions, including structured exercise programs and recreational activities, and only considered articles published in English.

## 3. Results

Only five articles met the inclusion criteria for the Systematic review; therefore, the remaining articles were included in the narrative review.

### 3.1. Study Selection

**Figure 1:**
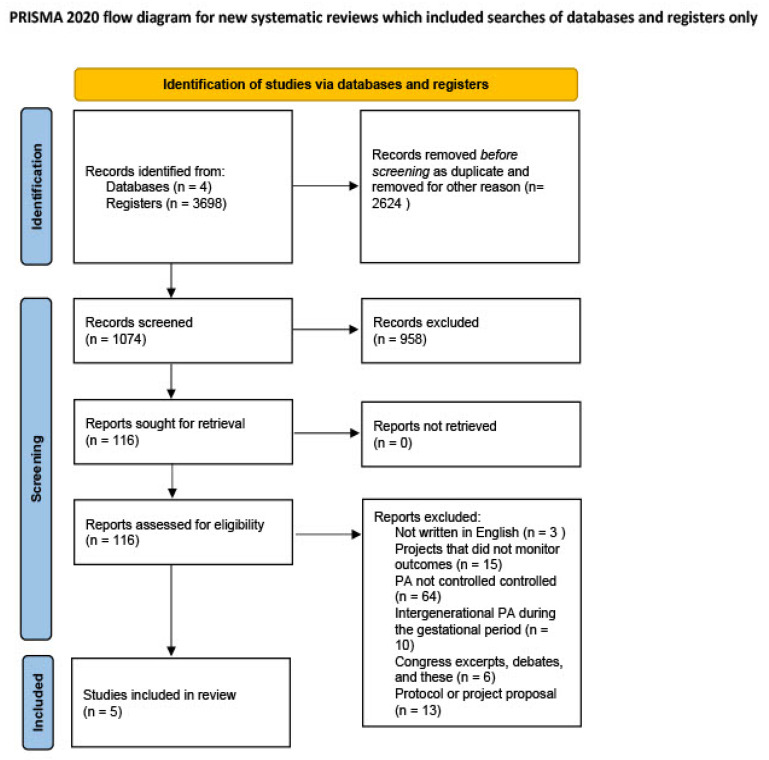
Flowchart.

The database search yielded 3698 potential records. After duplicate removal, 1074 potential records were evaluated to be included in the present systematic review. After screening based on title and abstract, 116 potential articles remaining. The full text of these 116 articles were finally evaluated to verify whether these articles met the inclusion and exclusion criteria identified for the present systematic review. Only five articles resulted suitable to be included in the review. This process adheres to the PRISMA statement, and the flowchart of the selection process was reported in Figure 1.

### 3.2. Characteristics of the Participants

The characteristics of the included studies are presented in Table 2. A total of 1037 participants were included in the analysis.

The studies were conducted on a healthy population of children, adolescents, adults, and elderly individuals belonging to both genders.

### 3.3. Risk of Bias Assessment

**Figure 2:**
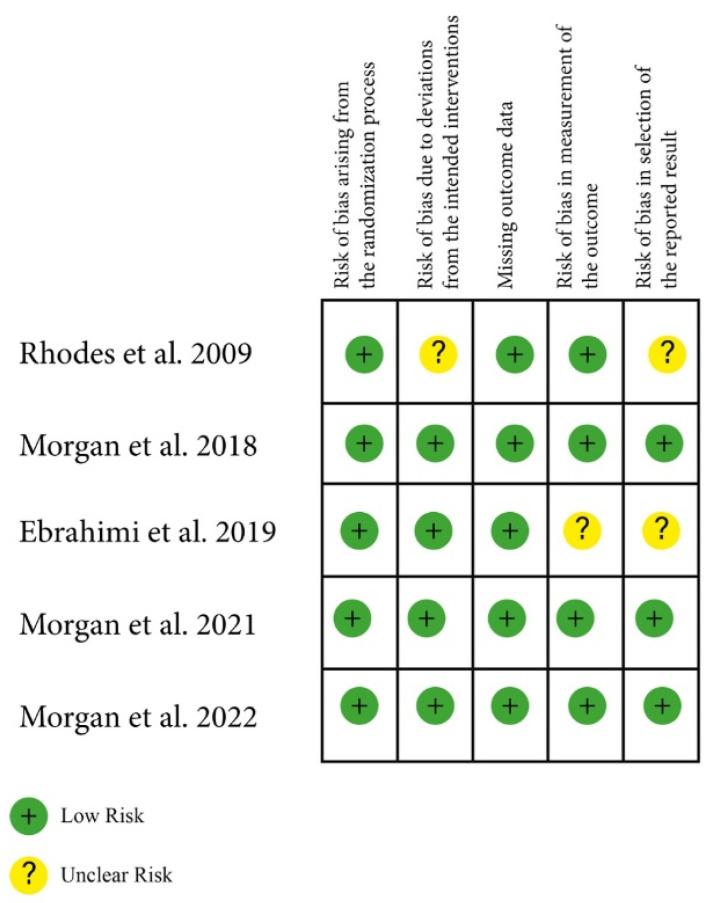
Risk of Bias Assessment [18,19,20,21,22].

All studies were considered to have a low risk of bias for the “randomization process” domain since we only included randomized studies (Figure 2). Inversely, the domain “Deviations from intended interventions” was rated to be at high risk of bias in all studies due to the impossibility of blinding participants and people delivering the intervention to group assignment in the exercise interventions. 

The Cochrane RoB2 tool includes an assessment of double blinding for randomization. However, given the nature of the studies under review, and since participant blinding is not achievable in such studies, this parameter was not considered.

### 3.4. Characteristics of the Five Intervention Articles Included in the Systematic Review

The intervention, ranging from 2 to 25 weeks, included the following: (a) guides or programs to promote PA and an active lifestyle with tasks to be completed at home; (b) intergenerational sport activities (yoga, tai-chi, dances); (c) questionnaires and monitoring tools.

This analysis of the five RCTs provides in-depth insight into the methodologies, objectives, and outcomes. Among the five studies included, three [19,21,22] were conducted by the same research team. Despite differences in study populations and interventions, some significant similarities emerge.

A key element is the intervention-based approach, where Rhodes et al. [18] focused on implementing a family planning strategy, while Morgan et al. [19] centered the DADEE Trial on engaging fathers as role models for their daughters. Both studies achieved significant increases in family PA, highlighting the effectiveness of targeted interventions in positively influencing behaviors. Differences in outcome measures are evident, reflecting the specific goals of each study. While Rhodes et al. [18] focused on the frequency and duration of family PA, Morgan et al. [19] included measures such as fundamental motor skills and screen time. This variety of measures provides a more comprehensive perspective on the impacts of interventions, allowing for a deeper assessment of family dynamics and lifestyles.

Ebrahimi et al. [20] introduced a unique perspective with a study on the elderly, comparing the effects of yoga exercise programs and intergenerational interaction on mental health. This perspective offers a broader understanding of the benefits of family interventions, extending beyond the parent–child context. However, limitations in geographical representativeness and sample size may affect the generalizability of the results.

Morgan et al. [22] further contributed by exploring the efficacy of the DADEE program and the “Healthy Youngsters, Healthy Dads” Program. While the DADEE Trial showed a significant increase in PA for fathers and daughters, the other study focused on fathers and preschool-aged sons. Both interventions demonstrated a positive impact, such as fundamental motor skills in preschool-aged children.

Common limitations include the use of self-reported measures, the potential for sampling bias, and the short duration of some studies. The pedometer-based method for measuring PA, might not fully capture the complexity of activity patterns. Additionally, individuals’ adherence to interventions and variable engagement might have influenced outcomes.

Emphasizing the importance of considering family dynamics and specific contexts enhanced the impact of intergenerational programs on health and well-being.

## 4. Discussion

Despite the significant number of studies on intergenerational activity that highlighted positive outcomes in terms of improvement in psychological well-being and health, few studies achieve these results through scientifically conducted analysis (sufficient sample size, the presence of a control group, objective assessment of results, often self-reported outcomes], making the results of the excluded studies questionable from a scientific perspective. Among the five studies included, three [19,21,22] were conducted by the same research team, which may limit the generalizability of the findings due to potential bias. The only five selectable RCTs, including three that come from the same research group, highlight the need for new studies to further explore the topic of intergenerational training, adopting a rigorous scientific approach. Nevertheless, the five RCTs provide an in-depth insight into methodologies, objectives, and outcomes in the contexts of intergenerational PA and health interventions. 

### 4.1. Considerations of the Five Articles in the Systematic Review

The key element of the five RCTs was the effects of PA shared among family members: fathers–children, grandparents–grandchildren. Significant enhancements in motor skills, such as those resulting from intergenerational interventions, were demonstrated across all studies examining children. It is interesting that interventions with children consistently produced improvement effects, whereas interventions with adults generally resulted in the maintenance of physical efficiency or mild enhancements of abilities.

We can underline that the intergenerational PA intervention amplified a process of maturation in children that was naturally moving in the same direction of improvement and development [18]. Regarding adults and older adults, the natural process of deterioration was countered and delayed by the intervention of physical and relational activities, resulting in a slight improvement or at least a deceleration of physical and cognitive decline [20]. In addition to fundamental motor skills, Morgan et al. [22] included measures such as screen time reduction.

The selected articles highlighted the pairing of adults and children (four articles) and the pairing of older adults and children (one article). Parents, as adults, act as “gatekeepers” for their children, increasing fidelity and adherence to PA in their children by following guidelines related to a behavioral plan strengthening family bonds and overall family health.

Moreover, families’ involvement in intergenerational PA programs predicted family activity levels, promoting good lifestyle behaviors and good interactions between family members. This partnership could be a key to creating self-sustaining, cost-effective, and successful programs.

Rhodes et al. [18] highlighted the importance of promoting a self-regulatory approach within the family context encouraging the personalization of interventions through an autonomous behavioral plan (“what, when, where, who”). This characteristic can help overcome barriers (laziness, lack of time, forgetfulness) that may hinder the development of these health-promoting behaviors.

Ebrahimi et al. [20] examined the effects of intergenerational activity involving more distant generations, with grandparents and grandchildren. Integration of PA between grandparents and grandchildren may be more challenging to implement; nevertheless, it could be considered that grandparents are retired and have more opportunities to dedicate time to these activities without obvious time constraints.

The utilization of structured intergenerational training plans, facilitated by informative guides, contributes to effective activity schedules and enhances parents’ capacity for time management, which leads to an increase in PA levels and improves family relationships [22].

### 4.2. Considerations of the Articles in the Narrative Review

#### Demographic Characteristics: Age, Gender and Social Economic Status

The age of participants in the selected studies showed a wide range of group combinations: older adults with young adults [20], grandparents and grandchildren [23,24], and parents and children [21,22,25]. The different combinations depend on the specific objectives to be achieved: “parents and children” represents a viable solution for achieving the recommended daily activity levels outlined by the WHO, thereby overcoming time-related barriers that individuals may encounter in terms of allocating time for their own PA or that of their children. 

Family participation in PA is seen as both a way to facilitate engagement in activities and a means to strengthen family bonds and overall health. Interactions between “young and older adults” help reduce negative self-perception among the elderly and support them in motor or creative tasks [1,26]. The most common pairing in studies is “older adults with children” or “grandparents with grandchildren.” Older adults typically move slower, have longer reaction times, and may face physical limitations, affecting how children interact with them. Conversely, children often need to adapt their motor patterns when engaging with older adults, presenting new challenges and experiences compared to interacting with peers. Children may need to adapt their movement direction, speed, and precision when interacting with older adults, enhancing their body control and movement awareness. Conversely, the energy of children motivates older adults, providing new incentives for engagement [23].

Participation in intergenerational projects has been higher among women than men [23,24,27]. It is easy to think that the care of children and grandchildren is traditionally associated with women, so the time dedicated to grandchildren is greater among women. 

However, recent studies on promoting PA among the elderly have revealed that women’s participation in PA initiatives for the elderly is higher than men [28,29,30].

Socioeconomic status influences intergenerational program participation: it is easier for individuals with less-busy work schedules to allocate their leisure time to functional exercise for their health or to provide their children/nephews with maturation and health opportunities, promoted by PA and sports [25]. Different elements across different ecological levels have been identified as significant in comprehending the levels of PA both among young and old individuals. As highlighted in several studies, older adults who engaged in intergenerational activities tended to have a high educational level [high school or bachelor’s degrees), and in many cases, were retired individuals [31].

### 4.3. Types of Intergenerational Programs for Different Goals

The review of the selected studies indicates that some activities are more effective for intergenerational approaches, while others serve different purposes better. Therefore, the choice of protocols should be tailored to the specific objectives intended [20].

Kim’s study [32] found that participants in intergenerational yoga or tai chi showed better relational and well-being outcomes, while the peer-based control group excelled in reducing anxiety and sleep disorders. Although physical activity (PA) programs for the elderly often have specific requirements that may not suit children’s activities, intergenerational interactions enhance the enjoyment and motivation of older adults more effectively than peer-only group exercises [23]. Additionally, taekwondo promotes principles like courtesy, mutual respect, and self-discipline, along with diverse movements, improving participants’ physical abilities [33].

Psychomotor exercises that involve proprioceptive, sensory, motor, and social tasks—sometimes using tools and organized intergenerationally—benefit child development and counteract sensory-motor decline in the elderly. Mosor [27] found that such programs led to increased spontaneous intergenerational interactions and higher engagement among older adults.

Intergenerational sports activities like dance enhance socialization, sleep quality, and reduce anxiety and depression [34]. Dance is also sustainable and feasible due to the availability of suitable environments, highlighting its significant benefits [26].

Additionally, multilevel interventions such as intergenerational activities including music, games, and educational initiatives, aimed at facilitating communication and interaction among participants, enhance children’s perception of older adults and may potentially alleviate elderly clinical depression [35].

Intergenerational programs that incorporate PA and technology for monitoring it have been shown to enhance technology skills in older adults through interactions with younger people. Participation in daily and healthcare activities with different generations offers benefits across various categories [24].

### 4.4. Positive Outcomes of Intergenerational PA Programs

#### 4.4.1. Motor Skills

Intergenerational interventions significantly improved children’s motor skills, including jump power and handgrip strength [23]. These interventions consistently benefited children, while in the elderly, they helped maintain physical efficiency or led to slight improvements. We can underline that the intergenerational PA intervention amplified a process in children that was naturally moving in the same direction of improvement and development. Regarding the elderly, the natural process of deterioration was countered and delayed by the intervention of physical and relational activities, resulting in a slight improvement or at least a deceleration of physical and cognitive decline. However, certain aspects, such as control of objects or jump height, showed only marginal improvements in both the samples [23]. These interventions likely did not focus on improving physical performance and may have lacked the necessary intensity, volume, and frequency to achieve performance-based improvements.

These findings emphasize the importance of aligning objectives with the structure of activities to achieve desired outcomes [25].

#### 4.4.2. Cognitive Outcomes

Engagement with children can stimulate the cognitive capacities of older adult participants and the maturation of children. Children and older adults will adjust their speed and range of movements to each other, thus enhancing their awareness of movements that require improved body control [23]. 

Involvement in activities with the younger generation challenges the mind and promotes executive functions, memory, and brain activity/volume, contributing to an overall enhancement of cognitive efficacy [36]. Krueger et al. [2] found that problem-solving skills and processing efficiency are more strongly correlated with supportive relationships from intergenerational activities than with information retention. This suggests that intergenerational activities offer cognitive enrichment, but only when sustained over time [37].

#### 4.4.3. Social and Well-Being Outcomes

The main advantages of intergenerational PA are oriented to the social and relational sphere. Research indicates that all groups involved in the program have witnessed improvements in social skills, psychological well-being, and the ability to sustain relationships [38]. Older adults enhance their self-esteem and the empowerment of their self-perception, reducing feelings of loneliness and depression [39], while children improve their socio-emotional development [40]. Increasing social interactions between children and seniors, during intergenerational activities, effectively reduces stereotypes and apprehensions, improving an environment where both generations can learn and grow together [26]. Educational opportunities for both age groups could inspire new life projects for older participants and exert a positive influence on their significance of life [24,27].

#### 4.4.4. Overcoming Social Barriers

Interactions spanning different generations transcend social barriers, creating an all-inclusive setting where generations can share experiences, mutually learn, and cultivate meaningful bonds [23].

Interactions with children, specifically, contribute to heightening the quality of life for elderly residents, amplifying psychosocial well-being alongside physical performance [25]. The utilization of structured intergenerational training plans, facilitated by informative guides, contributes to effective activity scheduling and enhances parents’ capacity for time management [18].

## 5. Limitations

Common limitations include the use of self-reported measures, the potential for sampling bias, and the short durations of some of the studies. The pedometer-based approach to measuring PA, used in the majority of the studies, may not fully capture the complexity of the activity patterns. Moreover, the outcomes may have been influenced by individuals’ adherence to interventions and their varying levels of engagement. Among the five studies included, three were conducted by the same research team, which may limit the generalizability of the findings due to potential bias. Limitations in geographical representation and limited sample sizes may affect the generalizability of the results. 

Feature research is required, using comparable models that enable the generalizability of findings, including RCTs, to generate the highest-quality evidence. Moreover, these results should be followed up to ensure that these new and healthier behaviors persist over time. 

## 6. Implications for Policy and Practice

Considering the positive outcomes for the elderly in promoting an active lifestyle and stimulating cognitive and physical abilities, as well as improving their emotional well-being and positive acceptance of the elderly among children, it is essential to promote these programs not only within schools, sports associations, and cultural clubs, but also within healthcare facilities and nursing homes. 

The first step should be a comprehensive understanding of the characteristics, needs, and expectations of both groups involved in intergenerational PA programs. Successively, identifying the most suitable and enjoyable PA for both groups, to promote adherence to the programs, whose effectiveness is ensured by long-term participation, as with all types of PA programs. 

The organization of workshops and preparatory sessions, taking into account the characteristics and needs of individuals of different ages, will contribute to a more effective approach to promoting relationships and interactions among participants [7]. It might be interesting to schedule follow-ups to ascertain whether the achieved results, such as the enhanced social–emotional well-being of both children and older adults, can be sustained over the long term. 

Intergenerational promotion efforts start with the widespread dissemination of these proposals and the need for adaptation for different contexts, as suggested by the obtained results. On the one hand, the dissemination program, supported by “train-the-trainers” initiatives, will enhance the effectiveness of the proposals in terms of promoting a more active lifestyle and improving intergenerational relationships. On the other hand, positive results will lead to government awareness and promotion of these initiatives, ensuring health improvement.

## 7. Conclusions

Intergenerational PA may offer benefits beyond exercise, stimulating cognitive abilities in older adults, and may aid children’s maturation. These interactions positively impact children’s socio-emotional development and can have lasting effects. For the elderly, these programs help mitigate age-related deficits, while children benefit from improved motor control and emotional well-being by adapting to their older counterparts. Different activities must be tailored to the characteristics and needs of different age groups to achieve specific goals. For the younger generation, this experience relocates the elderly from marginal figures to valuable sources of experiential and emotional knowledge. Shared physical activity between fathers and children, or grandparents and grandchildren, can facilitate overcoming the challenges of organizing daily life by promoting an active lifestyle habit among younger generations, ensuring healthy aging. If the self-regulation approach is used by families through the use of apps or guidelines, adults will be able to help both the elderly and children follow a protocol remotely, promoting digital literacy among both younger and older generations.

Grandparents play a crucial role as educational mediators, aiding in the integration of their grandchildren into diverse environmental dynamics.

## Figures and Tables

**Table 1:** PICOS Criteria.

Research Question	Population	Intervention	Control Group	Outcome
Description	Children, Adolescence, Adults, and older Adults	Participation in group physical activity (PA) and active involvement	Intervention vs. non-intervention groups	Measurement of behavior change and other health-related outcomes reported in the included studies
Inclusion Criteria	Randomized controlled trials (RCTs)	Protocols based on intergenerational physical activity	A description of the intergenerational physical activity protocol must be provided.	Studies can report other relevant outcomes that may have been impacted by health behavior change (e.g., cognitive benefits, motor, physical well-being, and social–emotional benefits, overcoming social barriers)

**Table 2:** Characteristics of the studies.

Title and Reference	Participants	Duration	Results
Pilot study of a family physical activity planning intervention among parents and their children [18]	N = 65	4 Weeks	Improved PA/minute × Week
Engaging Fathers to Increase Physical Activity in Girls: The “Dads And Daughters Exercising and Empowered” (DADEE) Randomized Controlled Trial [19]	N = 268	36 Weeks	Fathers and daughters improved physical activity levels (pedometer). Daughter improved skill proficiency.
Comparing the efficacy of yoga exercise and intergenerational interaction program on mental health of elderly [20]	N = 235	4 Weeks	Decreased anxiety Improved quality of sleep
Establishing Effectiveness of a Community-based, Physical Activity Program for Fathers and Daughters: A Randomized Controlled Trial [21]	N = 125	36 Weeks	Improved step count Reduction in fathers’ and daughters’ screen time
Impact of the ‘Healthy Youngsters, Healthy Dads’ program on physical activity and other health behaviors: a randomized controlled trial involving fathers and their preschool-aged children [22]	N = 344	12 Weeks	Improved step count
